# New PAR1 Agonist Peptide Demonstrates Protective Action in a Mouse Model of Photothrombosis-Induced Brain Ischemia

**DOI:** 10.3389/fnins.2020.00335

**Published:** 2020-05-19

**Authors:** Maksim Galkov, Ekaterina Kiseleva, Mikhail Gulyaev, Maria Sidorova, Liubov Gorbacheva

**Affiliations:** ^1^Faculty of Biology, Lomonosov Moscow State University, Moscow, Russia; ^2^Electrophysiology Laboratory, Translational Medicine Institute, Pirogov Russian National Research Medical University, Moscow, Russia; ^3^Department of Cell Biology, Koltzov Institute of Developmental Biology of Russian Academy of Sciences, Moscow, Russia; ^4^Faculty of Fundamental Medicine, Lomonosov Moscow State University, Moscow, Russia; ^5^Laboratory of Peptide Synthesis, Institute of Experimental Cardiology, National Medical Research Center for Cardiology of Russian Ministry of Health, Moscow, Russia

**Keywords:** protease-activated receptor 1 (PAR1), PAR1 agonist peptide, protein C, β-arrestin-2, photothrombosis-induced focal ischemia

## Abstract

Protease-activated receptors (PARs) are involved not only in hemostasis but also in the development of ischemic brain injury. In the present work, we examined *in vivo* effects of a new peptide (AP9) composing Asn^47^-Phen^55^ of PAR1 “tethered ligand” generated by activated protein C. We chose a mouse model of photothrombosis (PT)-induced ischemia to assess AP9 effects *in vivo*. To reveal the molecular mechanism of AP9 action, mice lacking β-arrestin-2 were used. AP9 was injected intravenously once 10 min before PT at doses of 0.2, 2, or 20 mg/kg, or twice, that is, 10 min before and 1 h after PT at a dose of 20 mg/kg. Lesion volume was measured by magnetic resonance imaging and staining of brain sections with tetrazolium salt. Neurologic deficit was estimated using the cylinder and the grid-walk tests. Blood–brain barrier (BBB) disruption was assessed by Evans blue dye extraction. Eosin-hematoxylin staining and immunohistochemical staining were applied to evaluate the number of undamaged neurons and activated glial cells in the penumbra. A single administration of AP9 (20 mg/kg), as well as its two injections (20 mg/kg), decreased brain lesion volume. A double administration of AP9 also reduced BBB disruption and neurological deficit in mice. We did not observe the protective effect of AP9 in mice lacking β-arrestin-2 after PT. Thus, we demonstrated for the first time protective properties of a PAR1 agonist peptide, AP9, *in vivo*. β-Arrestin-2 was required for the protective action of AP9 in PT-induced brain ischemia.

## Introduction

Vessel wall damage and hemostatic balance disturbances lead to changes in tissue blood flow and, as a result, to the development of ischemic conditions (Khoshnam et al., 2017). Activation of endothelial cells is a key factor for initiating thrombus formation. A wide range of receptors, which regulate the state of the endothelium and platelets, is involved not only in hemostasis but also in the development of ischemic brain injury. Protease-activated receptors (PARs) are ones of these receptors. PARs belong to the family of G-protein-coupled receptors (GPCRs) and regulate cellular and physiological processes in normal and pathological conditions (Ossovskaya and Bunnett, 2004; Coughlin, 2005).

The mechanism of PAR activation was revealed and thoroughly studied for PAR1 (Vu et al., 1991) and other PAR subtypes (Coughlin, 2005). Proteases cleave the receptor at the specific site of the exodomain that results in formation of a novel N-terminus (“tethered ligand”). This N-terminal sequence interacts with the second extracellular loop and activates the receptor (Ossovskaya and Bunnett, 2004; Coughlin, 2005). It was shown that serine proteases of the blood coagulation system, thrombin and activated protein C (APC), exert multidirectional effects during excitotoxicity and neuroinflammation via PAR1 activation (Suo et al., 2004; Gorbacheva et al., 2008; Griffin et al., 2015).

Thrombin demonstrates procoagulant effects and increases expression of proinflammatory and proapoptotic factors in the nervous tissue (Suo et al., 2004; Coughlin, 2005). APC, in contrast to thrombin, protects neurons and endothelium, stabilizes the endothelial barrier, and controls cytokine gene expression in brain injury (Riewald and Ruf, 2005; Minhas et al., 2009; Zlokovic and Griffin, 2011; Griffin et al., 2015; Roy et al., 2016). Earlier we have shown PAR1-dependent antiapoptotic effects of APC on cultivated hippocampal neurons during glutamate excitotoxicity (Gorbacheva et al., 2009, 2010; Savinkova et al., 2014).

It was found that PAR1 activation initiates multidirectional pathways through both G-proteins (Traynelis and Trejo, 2007) and β-arrestins in cultured endotheliocytes (Soh and Trejo, 2011; Roy et al., 2016). The “molecular ensemble” around the receptor and its pattern of phosphorylation by GPCR kinases provide this signal dualism called “biased agonism” (Premont and Gainetdinov, 2007; Reiter et al., 2012). Cytoprotective signaling by APC involves a novel cleavage of PAR1 N-terminal domain at Arg^46^–Asn^47^ differing from that of thrombin (Arg^41^–Ser^42^) and β-arrestin-2-dependent pathway activation (Soh and Trejo, 2011; Roy et al., 2016). β-arrestin recruitment and activation of the Dvl-2 scaffold initiate APC-induced cytoprotection mediated by Rac1. In this case, activated Dvl-2 may provide a platform that recruits effector proteins, such as Rac1-specific guanine nucleotide exchange factors to promote Rac1 activation (Soh and Trejo, 2011). For APC-induced β-arrestin-2-dependent signaling, the colocalization of PAR1 with the endothelial protein C receptor and thrombomodulin in caveolae is required (Griffin et al., 2015; Roy et al., 2016).

The unique mechanism of PAR activation makes it possible to apply peptide analogs of the “tethered ligand” as receptor agonists (Mao et al., 2008; Ramachandran and Hollenberg, 2008; Mosnier et al., 2013). In the present work, we used a nine-amino-acid peptide (AP9, NPNDKYEPF-NH_2_), which mimics PAR1 N-terminus beginning at residue Asn^47^ and generated by APC cleavage at Arg^46^. The protective effects of AP9 via PAR1 activation were confirmed *in vitro* using primary cultures of neurons, mast cells (Savinkova et al., 2014; Babkina et al., 2016), and human keratinocytes (Kiseleva et al., 2014). Moreover, it was previously shown that another PAR1 peptide agonist, TR47, which also formed after PAR1 cleavage at Arg^46^, demonstrates the general activity profile of APC (Mosnier et al., 2013). Taken together, these data make it possible to hypothesize that AP9 will demonstrate APC-like protective properties, which are expected to be β-arrestin-2-dependent, in ischemic conditions *in vivo*. To test that hypothesis, we used a model of photothrombosis-induced (PT-induced) focal brain ischemia (Lee et al., 2007; Galkov et al., 2020) and different schemes of AP9 intravenous administration.

PT model based on the generation of reactive oxygen species under the action of light induces endothelium damage and triggers thrombogenesis (Lee et al., 2007). PT-induced pathological processes are similar to endogenously formed ones (Kim et al., 2001; Schroeter et al., 2002; Li et al., 2014; Kang et al., 2015; Cotrina et al., 2017). Moreover, PT-induced focal ischemia is characterized by low invasiveness and high reproducibility of the damage volume (Fluri et al., 2015), as well as the precise localization of lesion area in the cerebral cortex (Harrison et al., 2013).

In the present work, we examined the potential effects of PAR1 agonist peptide, AP9, in the mouse model of PT-induced brain ischemia. The role of β-arrestin-2-signaling in intracellular pathways triggered by AP9 was also revealed.

## Materials and Methods

### Animals

Male mice weighing 25–30 g and aged 2.5–3 months were used in the study. Animals were obtained from the nursery for laboratory animals of Shemyakin-Ovchinnikov Institute of Bioorganic Chemistry (Russian Academy of Sciences, Moscow region) and kept under standard conditions (temperature: 22°C, light cycle: 12 h) with unlimited access to water and food. We used BALB/c mice, β-arrestin-2-knockout mice (β-arrestin-2-/- group) kindly provided by Prof. R.R. Gaynetdinov, and animals of genetically related C57BL/6 strain (wild type) (Bohn et al., 1999) with full β-arrestin-2 expression. The total number of animals used was 177 (141 BALB/c mice, 20 C57BL/6 mice, and 16 knockout animals). Six BALB/c mice and one C57BL/6 mouse died during PT induction.

All animals of each strain were randomized into several groups (control groups and groups treated with AP9 at different doses). The randomization scheme was generated using the website Randomization.com^1^. The number of animals in each experimental group (*n*) is indicated in section “Results” (*n* = 4–10). All experiments were performed according to the Directive 2010/63/EU of the European Parliament and the Council of the European Union. Our experimental protocols were approved by the Bioethics Committee of Lomonosov Moscow State University (protocol no. 2018-10-25-93-0-3). All measurements were carried out in a blinded manner. A researcher did not know which group the animals belonged to during the processing of images and video records, as well as during Evans blue dye extraction.

### Mouse Genotyping

Modification of the β*-arrestin-2* gene in mice carried out by homologous recombination (Bohn et al., 1999) was confirmed by polymerase chain reaction (PCR). Genomic DNA was isolated from lysates of tail fragments of wild-type and β-arrestin-2-knockout mice by an Extract DNA kit (Eurogen, Russia). PCR was performed using Taq DNA polymerase and standard set of reagents (Thermo Fisher Scientific, United States). During PCR, three primers were used (Eurogen, Russia): primers flanking the insertion site of the genetic construct (forward 5’-GATCAAAGCCCTCGATGATC-3’ and reverse 5’-ACAGGGTCCACTTTGTCCA-3’), as well as the primer complementary to donor DNA sequence in the β*-arrestin-2* gene (forward 5’-GCTAAAGCGCATGCTCCAGA-3’). PCR products were separated on a 2% agarose gel with the addition of ethidium bromide. DNA ladder NL002 (Eurogen, Russia) was used as markers; visualization was performed using UV light in a transilluminator. A fragment of 400bp in length indicated the presence of a knockout allele, a 600-bp fragment indicated a wild-type allele. Animals homozygous for the inactivated allele were selected in the experiment.

### Western Blotting

#### β-Arrestin-2 Expression Level

Overanesthetized gene knockout and wild-type animals were transcardially perfused with heparinized saline (40 units/ml). Fragments of the cerebral cortex were isolated on ice and lysed in RIPA buffer and protease inhibitors (Sigma-Aldrich, United States). Samples containing 20 μg of protein were separated by 10% SDS-PAGE, transferred to nitrocellulose membranes, and blocked with 5% skim milk in TBS-Tween 20 (0.05%) for 1 h. The blot was developed by incubation with antibodies against β-arrestin-2 (1:1,000; ab206972, Abcam, United Kingdom) and β-actin (1:1,000; ab8227, Abcam, United Kingdom) overnight at 4°C, followed by incubation with horseradish peroxidase-conjugated anti-rabbit IgG antibodies (1:3,000; sc-2357, Santa Cruz Biotechnology, United States) for 1 h at room temperature. The presence of the target proteins on the blots was recorded using chemiluminescent ECL kit (Thermo Fisher Scientific, United States) and ChemiDoc MP Imaging System (Bio-Rad Laboratories, United States).

#### GFAP Expression Level

Overanesthetized control and AP9-treated mice were transcardially perfused 96 h after PT. After decapitation, the brain was isolated on ice, the cortex was separated, and the damaged brain tissue in the ipsilateral hemisphere (IH), as well as in the contralateral hemisphere (CH), was isolated by a circular knife 4 mm in diameter. Seventy micrograms of protein was applied into one gel pocket. Western blotting was carried out as described above. Antibodies against GFAP (1:5,000; ab7260, Abcam, United Kingdom), GAPDH (1:5,000; ab181603, Abcam, United Kingdom), and horseradish peroxidase-conjugated anti-rabbit IgG antibodies (1:3,000; sc-2357, Santa Cruz Biotechnology, United States) were used. Quantitative image processing was performed using the Image Lab software (United States). The data were presented as a relative GFAP level taken in CH for 1.

### Induction of Ischemic Stroke

Ischemia was induced by photothrombosis (Lee et al., 2007; Galkov et al., 2020). In brief, the mixture of Zoletil 100 (Virbac Sante Animale, France) and Xyla (Interchemie werken “De Adelaar” B.V., Netherlands) at doses of 35 and 3.5 mg/kg (i.p.), respectively, was chosen for anesthesia. Rose Bengal dye (0.15%; Sigma-Aldrich, United States) was injected through a catheter placed into the jugular vein of animals at a dose of 10 mg/kg. An area of 3 mm in diameter localized with the help of a light-tight mask in the sensorimotor area of the cortex of the left hemisphere (2.2 mm lateral to the midline with the epicenter at *bregma*, Franklin and Paxinos, 2001) was illuminated with green laser (λ = 532 nm; Viacho, China) for 5 min. Illuminance on the skull surface was 55–60 lx (Galkov et al., 2020). Mice were sacrificed by an overdose of anesthesia 24 or 96 h after PT.

Previously, we tested the ischemic model and showed that there was no tissue damage in sham-operated mice (administration of saline instead of Rose Bengal dye) according to MRI and 2,3,5-triphenyltetrazolium chloride (TTC) staining data (Supplementary Figures 1A,B). We also showed that there was no Evans blue dye leakage in the IH of sham-operated animals (Supplementary Figure 1C).

### Assessment of Neurological Status

The cylinder and the grid-walk tests were performed before PT induction and for 4 days after it according to the standard protocol (Baskin et al., 2003). The number of cases, when the mouse forelimb contralateral to the damaged hemisphere went through grid holes (stepping errors), and the number of steps performed by that limb were video registered for 5 min. The animals, which performed less than 100 steps, were excluded from the experiment. Sensorimotor deficit in the cylinder test was calculated according to the following formula: asymmetry index = (*t*_after_/*t*_before_) × 100%, where *t*_after_ and *t*_before_ were the relative contact time of the fore contralateral limb (the contact time of the ipsilateral limb was taken as 100%) after and before PT, respectively. We processed not less than 10 explorations of cylinder walls by each animal in the video records.

### Measurement of Ischemic Damage Volume

Lesion volume was assessed using MRI and staining of brain sections with TTC. MRI studies were performed 24 and 96 h after PT on a 7.05-T MR scanner BioSpec 70/30 USR (Bruker, Germany) equipped with fast gradients (105 mT/m/ms slew rate). We obtained T_2_-weighted MR images using the 2D RARE method with the following scanning parameters: TR/Teeff = 3,000/50 ms; number of slices: 16; slice thickness: 0.4 mm; rare-factor: 4; in-plane resolution: 100 × 100 m; total acquisition time: 9 min 00 s. To measure lesion volume using TTC staining, the animals were decapitated 24 h after thrombosis and the brain was removed. The damage volume was assessed according to the standard protocol as described previously (Galkov et al., 2020).

Digital images were processed in the ImageJ software (NIH, United States). The damage volume was calculated considering an increase in IH size as a result of edema compared with CH (Pialat et al., 2007). Data were presented as relative lesion volume. That parameter in control mice was set as 100%.

### Measurement of Blood–Brain Barrier Permeability

Evans blue dye (Sigma-Aldrich, United States) was injected intravenously through a catheter immediately after laser irradiation (80 mg/kg, 2.2% solution). Mice were transcardially perfused with heparinized saline (40 units/ml) 24 h after PT induction. To assess blood–brain barrier (BBB) permeability, dye extraction and determination of its content in both hemispheres were performed as previously described (Kim et al., 2001). The data were expressed as relative Evans blue content in IH. That parameter in CH was taken as 100%.

### Tissue Processing and Histological Analysis

Overanesthetized animals were transcardially perfused with 0.1 M phosphate buffer saline (PBS) and 10% formalin 24 or 96 h after PT induction. For immunohistochemistry, the brain was removed, postfixed for 24 h, washed in PBS, and sequentially incubated in 10, 20, and 30% sucrose at room temperature. Then the brain was embedded in an OCT embedding matrix (CellPath, United Kingdom), frozen, and stored at −80°C. For hematoxylin-eosin staining, the brain was removed, fixed in 10% neutral-buffered formalin for 24 h, embedded in paraffin, and cut into 5-μm sections that were stained.

For each animal, cell counting was performed in two sections of the central part of the lesion spaced apart from each other by at least 200 μm. The cells were counted in four non-overlapping fields (300 × 220 μm) of each section both in IH and in CH. The fields of view evenly covered the penumbra region and were located at the outer boundary of the ischemic core in the damaged hemisphere. In CH, we chose mirror areas of the cortex for cell counting (Galkov et al., 2020). Images were processed in the ImageJ software; the obtained data were averaged for one animal.

### Hematoxylin-Eosin Staining

The proportion of undamaged neurons was evaluated 24 h after thrombosis. The neurons that were not characterized by nuclear pyknosis, karyorrhexis, karyolysis, hyperchromia, swelling, and alteration of normal cell shape (Hu et al., 2002; Song et al., 2012; Galkov et al., 2020) were considered as undamaged cells. The data were presented as a relative number of undamaged neurons in the penumbra (similar indicator in CH was taken as 100%).

### Immunohistochemistry

The number of activated glial cells was evaluated 96 h after thrombosis. Coronal cryosections with 15-μm thickness were immunostained with antibodies against GFAP (1:500; ab10062, Abcam, United Kingdom) and Iba-1 (1:500; 019-19741, Wako, Japan). Secondary antibodies were conjugated with fluorophore Alexa Fluor 594 [anti-mouse IgG (1:1,000; R37121) or anti-rabbit IgG (1:1000; A-11037), Thermo Fisher Scientific, United States]. Cell nuclei were stained with DAPI (Biotium, United States). Images were obtained using confocal laser scanning microscope LSM 710 (Zeiss, Germany). The number of GFAP-positive cells (in two to four layers of cerebral cortex, Cotrina et al., 2017) and Iba-1-positive cells was counted. The data were presented as a relative value taken in CH for 1.

### AP9 Structure and Its Administration Scheme

Protease-activated receptor 1 agonist peptide (AP9), consisting of nine amino acids (NPNDKYEPF-NH_2_), was synthesized in the Laboratory of Peptide Synthesis of the National Medical Research Center for Cardiology (Moscow) using Fmoc solid-phase synthesis. The homogeneity of the peptide was confirmed by the data of analytical HPLC.

Initially, we used three doses of AP9 (0.2, 2, and 20 mg/kg) dissolved in saline at concentrations of 0.06, 0.6, and 6 mg/ml, respectively. AP9 administration was carried out intravenously 10 min before PT induction (Abdelkarim et al., 2001) through a catheter (as a bolus). In the next experimental series, the injection of AP9 at a dose of 20 mg/kg, which showed maximal efficiency, was performed 10 min before thrombosis and 1 h after PT.

### Statistical Analysis

The statistical analysis was performed using the GraphPad Prism 6 software (GraphPad Software Inc., United States). The D’Agostino–Pearson normality test was used to determine correspondence of sample data to normal distribution (*p* > 0.1). The data are expressed as mean ± SEM. The differences between groups were considered as statistically significant at *p* < 0.05. The number of animals (*n*) and statistical criteria are shown for each experiment. The power analysis performed using the STATISTICA software (StatSoft Inc., United States) showed that the statistical power was more than 0.8 in the case of detecting statistically significant differences between the data groups.

## Results

### Effect of AP9 on Brain Lesion Volume After Photothrombosis

**FIGURE 1:**
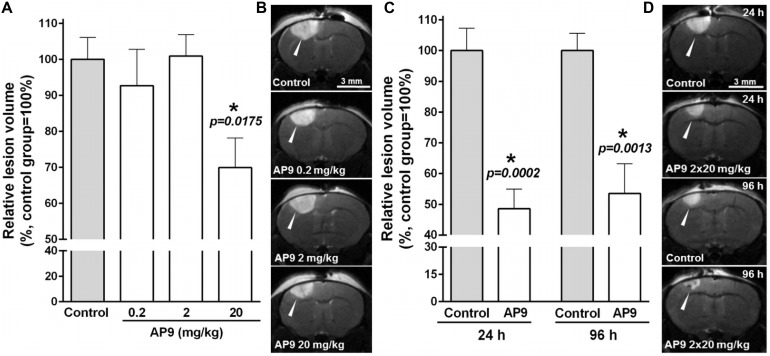
AP9 effects on brain lesion volume after photothrombosis. **(A,B)** A single administration of AP9 at doses of 0.2, 2, and 20 mg/kg: **(A)** relative volume of ischemic damage (one-way ANOVA with Dunnett’s correction for multiple comparisons) and **(B)** representative MR images at the level of *bregma* (damaged areas are marked with triangles) 24 h post thrombosis. **(C,D)** A double administration of AP9 at a dose of 20 mg/kg: **(C)** relative volume of ischemic damage (unpaired *t*-test) and **(D)** representative MR images at the level of *bregma* (damaged areas are marked with triangles) 24 and 96 h post thrombosis (*: as compared to the control group).

MRI was used to detect brain damage volume, which directly reflects the severity of ischemia. We found that AP9 effects were dose dependent. AP9 at a dose of 20 mg/kg (a single injection 10 min before PT) caused a statistically significant decrease in lesion volume, which amounted to 69.92 ± 8.21% (*n* = 7) in the AP9-treated group relative to control animals (100.00 ± 6.10%, *n* = 7) 24 h after PT induction. However, we did not observe the effect of AP9 at doses of 0.2 and 2 mg/kg on that parameter, which was 92.70 ± 10.10% (*n* = 4) and 100.90 ± 5.96% (*n* = 4), respectively (Figures 1A,B).

The repeated administration of the peptide (i.e., 10 min prior to and 1 h after PT) at a dose of 20 mg/kg enhanced the protective effect of its single injection and led to a statistically significant twofold decrease in lesion volume 24 h after thrombosis. The damage volume was 48.59 ± 6.42% (*n* = 7) in the AP9-treated group and 100.00 ± 7.27% (*n* = 7) in control mice. Moreover, we also evaluated lesion volume 96 h after thrombosis. It was 53.21 ± 9.72% in the AP9-treated group relative to the control value (100.00 ± 5.58%) (Figures 1C,D). Thus, MRI results indicated the preservation of the protective effect of double AP9 administration in the postischemic period.

### AP9 Did Not Affect Photothrombosis-Induced Morphological Changes in Neurons

**FIGURE 2:**
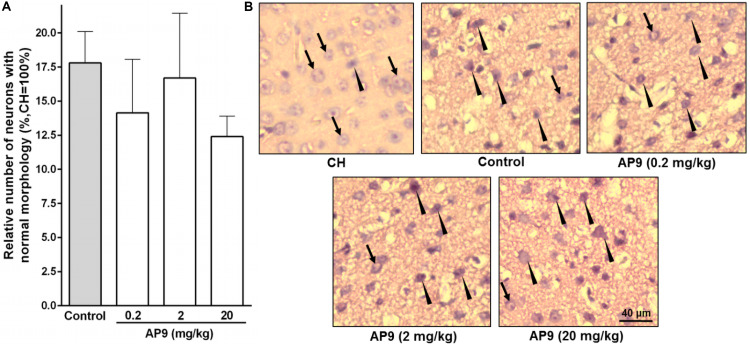
Morphological changes in neurons 24 h post thrombosis in control animals and AP9-treated mice (a single injection, 0.2, 2, and 20 mg/kg). **(A)** Relative number of undamaged neurons in the penumbra (one-way ANOVA). **(B)** Representative micrographs of the penumbral region in IH and a typical micrograph of the mirror region in CH (hematoxylin-eosin staining; arrows indicate cells with normal morphology, triangles indicate cells with signs of damage).

The pathological changes occurring after ischemia induction at the cellular level were assessed by hematoxylin-eosin staining of brain sections. We revealed a significant decrease in the number of neurons without morphological signs of damage in the penumbra compared with the similar areas in CH 24 h after PT (Figure 2).

A single injection of the peptide at all three doses used (0.2, 2, and 20 mg/kg) did not affect the proportion of undamaged neurons in IH compared with the control group. These portions were 14.14 ± 3.93% (*n* = 5), 16.70 ± 4.74% (*n* = 5), and 12.40 ± 1.50% (*n* = 7) at doses of 0.2, 2, and 20 mg/kg of AP9, respectively. In the control group of animals, the penumbral region contained 17.80 ± 2.30% (*n* = 8) neurons with no signs of damage (Figure 2).

**FIGURE 3:**
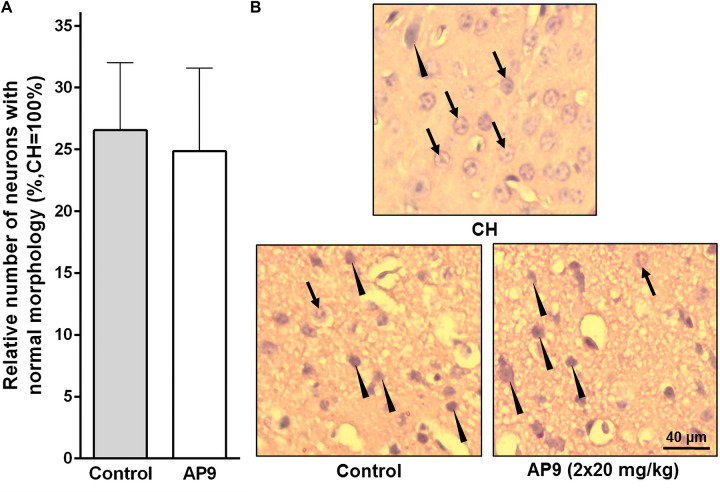
Morphological changes in neurons 24 h post thrombosis in control animals and AP9-treated mice (a double injection, 20 mg/kg). **(A)** Relative number of undamaged neurons in the penumbra (unpaired *t*-test). **(B)** Representative micrographs of the penumbral region in IH and a typical micrograph of the mirror region in CH (hematoxylin-eosin staining; arrows indicate cells with normal morphology, triangles indicate cells with signs of damage).

Two injections (10 min before and 1 h after PT) of AP9 at a dose of 20 mg/kg also did not change the relative number of undamaged neurons in IH. The penumbra was characterized by 26.56 ± 5.47% (*n* = 5) neurons of normal morphology in the control group of animals and 24.85 ± 6.74% (*n* = 7) in AP9-treated mice (Figure 3).

### AP9 Administration Did Not Change Photothrombosis-Induced Glial Cell Activation

According to published data, significant glial cell activation and proliferation are observed in the penumbra 96 h after PT induction. This is supported by enhanced expression of GFAP in activated astrocytes (Li et al., 2014; Cotrina et al., 2017) and Iba-1 in activated microglial cells/macrophages (Li et al., 2014).

**FIGURE 4:**
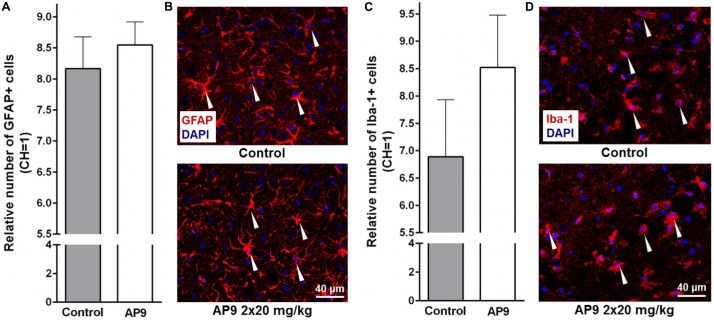
Glial cell activation 96 h post thrombosis in control animals and AP9-treated mice (a double injection, 20 mg/kg). **(A,B)** Immunohistochemistry data on GFAP staining: **(A)** relative number of activated astrocytes (unpaired *t*-test) and **(B)** representative microphotographs of the penumbra. **(C,D)** Immunohistochemistry data on Iba-1 staining: **(C)** relative number of activated microgliocytes/macrophages (unpaired *t*-test) and **(D)** representative microphotographs of the penumbra (GFAP- and Iba-1-positive cells are marked with triangles).

In the present study, we also observed a significant increase in the number of activated glial cells in IH compared with CH 96 h after thrombosis induction. However, the effect of AP9 double administration (20 mg/kg, 10 min before and 1 h after thrombosis) on these indices was not found. The relative number of activated astrocytes (GFAP-positive cells) in the penumbra was 8.17 ± 0.51 (*n* = 5) and 8.55 ± 0.37 (*n* = 6) in control and AP9-treated animals, respectively (Figures 4A,B). For activated microgliocytes/macrophages (Iba-1-positive cells), the values were 6.89 ± 1.04 (control group, *n* = 5) and 8.52 ± 0.96 (experimental group, *n* = 6) (Figures 4C,D).

Western blotting analysis confirmed that there was no difference between the control group and AP9-treated animals (20 mg/kg, a double administration) in GFAP expression 96 h after photothrombosis. The relative level of GFAP in control animals was 26.74 ± 3.01 in IH and 1.00 ± 0.09 in CH (*n* = 4). In AP9-treated mice, that parameter was 26.09 ± 2.30 and 1.00 ± 0.10 (*n* = 4) in IH and CH, respectively (Supplementary Figure 2).

### Effect of AP9 on BBB Disruption After Thrombosis

Extraction of Evans blue dye is an effective tool to estimate the functional state of the BBB after experimental ischemia (Belayev et al., 1996; Kim et al., 2001). According to published data, the maximal disruption of brain vessel integrity after PT is observed during the first day after its induction (Kang et al., 2015). For this reason, we performed dye extraction from IH and CH 24 h after surgery to assess the potential protective properties of AP9.

**FIGURE 5:**
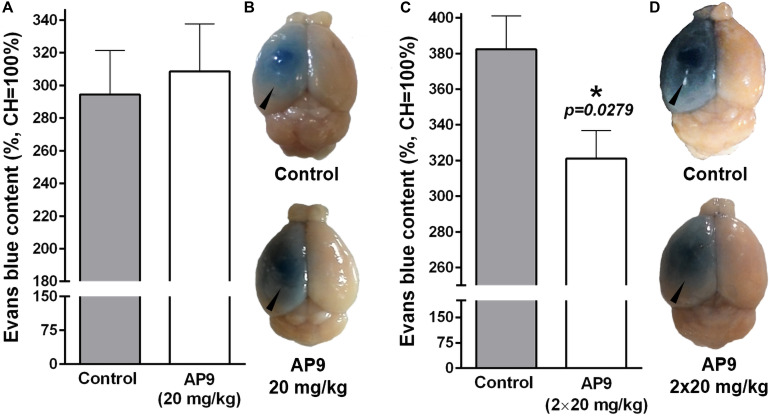
AP9 effects on PT-induced disruption of the BBB 24 h post thrombosis. **(A,B)** A single administration of AP9 (20 mg/kg): **(A)** relative Evans blue dye content in IH (unpaired *t*-test) and **(B)** representative photographs of the whole brains. **(C,D)** A double administration of AP9 (20 mg/kg): **(C)** relative Evans blue dye content in IH (unpaired *t*-test) and **(D)** representative photographs of the whole brains (triangles indicate damaged hemispheres).

A single administration of AP9 at a dose of 20 mg/kg did not affect Evans blue dye content in the damaged hemisphere. That index was 294.40 ± 26.88% (*n* = 5) and 308.60 ± 28.98% (*n* = 5) in control and AP9-treated mice, respectively (Figures 5A,B). However, a double injection of AP9 (20 mg/kg, 10 min before and 1 h after thrombosis) demonstrated the protective effect. The relative dye content in IH was 382.40 ± 18.67% (*n* = 6) in control animals. In mice treated with AP9, this parameter statistically significantly decreased down to 321.10 ± 15.69% (*n* = 7) (Figures 5C,D).

### Effect of AP9 on Photothrombosis-Induced Sensorimotor Deficit in Mice

Sensorimotor deficit developing in animals after experimental ischemia is a complex indicator of the severity of this pathology at the level of the whole organism. In the present study, we used the cylinder and the grid-walk tests, which previously showed high efficacy in detecting sensorimotor dysfunction in mice (Baskin et al., 2003; Galkov et al., 2020).

**FIGURE 6:**
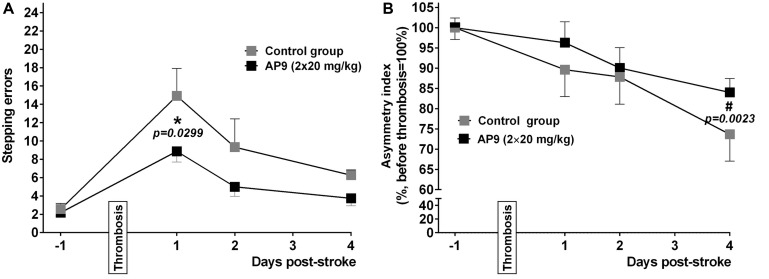
Effects of a double administration of AP9 (20 mg/kg, 10 min before and 1 h after PT) on PT-induced sensorimotor deficit in mice. **(A)** The grid-walk test data. **(B)** The cylinder test data (two-way ANOVA with Šidák correction for multiple comparisons, *: as compared to the corresponding control value, #: as compared to the value before PT).

We found that a double injection of AP9 at a dose of 20 mg/kg improved the neurological status of mice in both tests. In the grid-walk test, AP9-treated mice made fewer stepping errors (8.88 ± 1.18 errors/100 steps, *n* = 9) compared with the control group (14.92 ± 3.00, *n* = 7, two animals were excluded from the experiment due to the insufficient number of steps performed) 24 h after PT induction (Figure 6A). A statistically significant decrease in the asymmetry index measured in the cylinder test developed in the control group by 96 h after PT induction (73.64 ± 6.61 and 100.00 ± 2.93% 96 h after PT induction and prior to thrombosis, respectively; *n* = 7). This decrease was less expressed in the group subjected to AP9 treatment (84.00 ± 3.44 and 100.00 ± 2.39%, respectively; *n* = 8) (Figure 6B).

### Impact of β-Arrestin-2 Knockout on AP9 Protective Action in PT

**FIGURE 7:**
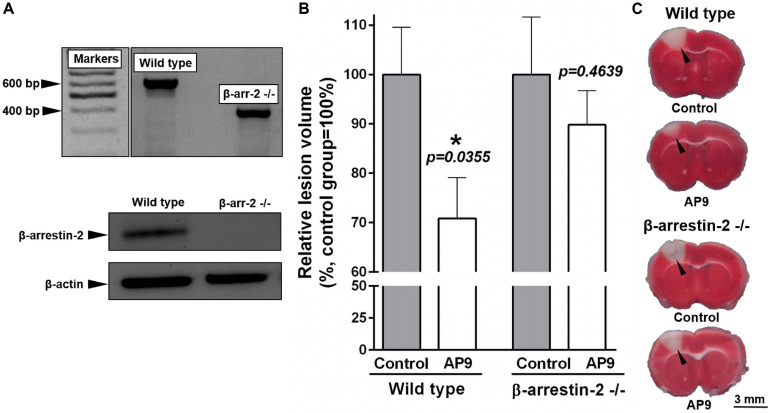
Effect of β-arrestin-2 knockout on AP9 protective action 24 h post thrombosis. **(A)** Confirmation of β*-arrestin-2* gene knockout [results of PCR analysis (above) and western blotting (below)]. **(B)** AP9 (20 mg/kg, a double administration: 10 min before and 1 h after PT) effect on relative lesion volume 24 h after PT in wild-type and gene-knockout mice (unpaired *t*-test for each group of animals). **(C)** Representative images of brain sections at the level of *bregma* (TTC staining; damaged areas are marked with triangles).

We used mice lacking β-arrestin-2 (Bohn et al., 1999) in order to reveal the contribution of this protein to the protective effects of AP9. According to the data obtained during the genotyping of animals by PCR, all β-arrestin-2-/- mice had knockout of both alleles of the target gene in contrast to wild-type animals. The results of western blotting completely confirmed the absence of β-arrestin-2 expression in knockout animals (Figure 7A).

We used TTC staining of brain sections to assess lesion volume. AP9 (20 mg/kg, 10 min before and 1 h after PT) significantly reduced the relative lesion volume in wild-type animals (C57BL/6 strain) 24 h after PT. This parameter was 70.80 ± 8.28% (*n* = 9) in the treated group and 100.00 ± 9.56% (*n* = 10) in control mice (Figures 7B,C). It was consistent with our MRI data from BALB/c mice. However, we did not find the protective effect of double AP9 injection in β-arrestin-2-/- mice. Lesion volume was 100.00 ± 11.63% (*n* = 8) in control animals and 89.82 ± 6.91% (*n* = 8) in AP9-treated mice (Figures 7B,C). Thus, β-arrestin-2 expression is required for AP9 protective action in PT-induced brain ischemia.

## Discussion

The involvement of hemostatic proteases in the regulation of brain ischemia-induced dysfunctions was demonstrated previously (Xi et al., 2003; Griffin et al., 2004; Suo et al., 2004; Sheehan and Tsirka, 2005). Data on the protective effects of APC and peptide analogs of PAR1 “tethered ligand” generated by APC cleavage at Arg^46^ are of particular interest. The effects of the nine-amino-acid peptide AP9 composed of Asn^47^–Phen^55^ of PAR1 “tethered ligand” in cultured neurons, mast cells, and keratinocytes were studied in our laboratory. We have shown using selective PAR1 blockers that AP9 increases the survival of neurons after their cocultivation with activated mast cells via PAR1 (Babkina et al., 2016). In addition, the peptide reduced neuronal death under excitotoxic conditions (Savinkova et al., 2014) and accelerated wound healing *in vitro* (Kiseleva et al., 2014). The similarity of PAR1-dependent action of AP9 and APC points to a possibility of AP9 effect implementation through APC-like pathways.

In the present study, we demonstrated for the first time the protective properties of AP9 in the mouse model of ischemic stroke. AP9 injected 10 min before PT at a dose of 20 mg/kg decreased brain damage volume. The double administration of AP9 (20 mg/kg) 10 min before and 1 h after PT, that is, within the “therapeutic window” (Danton and Dietrich, 2004), reduced not only lesion volume but also BBB disruption and neurological deficit in mice.

It is known that PAR1 is expressed by different components of the neurovascular unit: neurons (Junge et al., 2004; Gorbacheva et al., 2009), astrocytes (Bartha et al., 2000; Wang et al., 2002; Junge et al., 2004), and endothelial cells (Bartha et al., 2000). Accordingly, various types of cells can be considered as a potential target for AP9. Moreover, PT-induced BBB disruption increases the possibility of crossing the BBB by the peptide and leads to expansion of its cell target spectrum.

Nine-amino-acid peptide treatment decreased the lesion volume measured by MRI and Evans blue dye content in the damaged hemisphere after PT. MRI visualizes the brain areas characterized by edema formation (Hoehn et al., 2001), and Evans blue dye extraction allows us to evaluate the functional state of the BBB (Belayev et al., 1996). It indicates that the effects of AP9 are primarily due to the stabilization of the endothelial barrier function after PT-induced ischemia. Our results are in accordance with previous data on the PAR1 peptide agonist TR47, that is, the C-terminal fragment of PAR1 formed after cleavage of the receptor by APC at Arg^46^. TR47 inhibits endothelial permeability *in vitro* and *in vivo* vascular leakage in mice via APC-like activation of Rac1 (Mosnier et al., 2013).

The observed AP9 effects are similar to those described for APC. In cultured endotheliocytes, APC suppresses NF-κB-modulated genes and inhibits cytokine signaling. Moreover, APC modulates apoptosis pathways, including upregulation of the endothelial Bcl-2 homolog (A1), endothelial NO synthase, and the inhibitor of apoptosis (IAP) (Joyce et al., 2001; Riewald and Ruf, 2005). APC also activates the Ang1/Tie-2 axis (Minhas et al., 2009) and increases S1P synthesis (Feistritzer and Riewald, 2005; Finigan et al., 2005), decreasing disruption of the endothelial barrier. It is possible that all these effects can be triggered in brain endotheliocytes via activation of PAR1 by AP9.

**FIGURE 8:**
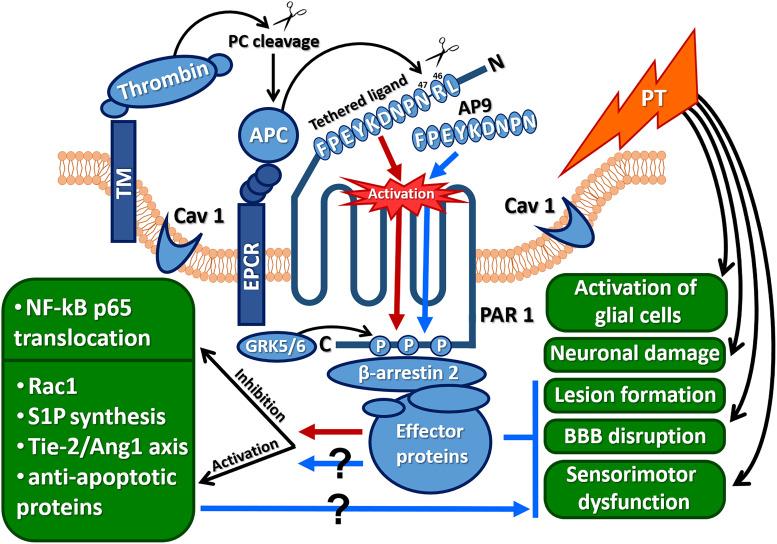
Protective effects of AP9 and possible peptide-triggered cytoprotective pathways similar to those induced by APC (Ang1, angiopoietin 1; AP9, nine-amino-acid peptide analog of PAR1 “tethered ligand”; APC, activated protein C; CAV1, caveolin-1; EPCR, endothelial protein C receptor; GRK 5/6, G-protein-coupled receptor kinases 5 and 6; NF-κBp65, p65 subunit of the nuclear factor κB; PAR1, protease-activated receptor 1; PT, photothrombosis; Rac1, GTPase; S1P, sphingosine-1-phosphate; Tie-2, angiopoietin receptor; red arrows indicate cytoprotective pathways triggered by APC, and blue arrows and lines indicate AP9 effects observed in the present research).

Despite the fact that AP9 beneficial effects were demonstrated on hippocampal neurons under excitotoxic conditions (Savinkova et al., 2014), as well as after their cocultivation with activated mast cells (Babkina et al., 2016), here, AP9 had no neuroprotective action according to histological analysis. It is possible that in addition to glutamate excitotoxicity and immune cell activation, other factors cause neuronal death under ischemic conditions *in vivo* (Durukan and Tatlisumak, 2007). Thus, a direct comparison of the results of *in vivo* and *in vitro* experiments is not applicable. Moreover, the lack of AP9 impact on neurons appears to be related to the method of peptide administration. Endothelial cells are expected to be a direct target of the peptide after its intravenous injection that is confirmed by AP9-mediated BBB stabilization after PT. However, according to the data on the sensorimotor tests, the peptide not only affects endotheliocytes but also indirectly protects other cell types via the regulation of PT-induced edema. Apparently, AP9 prevents cell damage in different components of the neurovascular unit via various cytoprotective pathways similar to APC (Figure 8). At the same time, the molecular mechanism of AP9 action needs further detailed study.

The APC-like mechanism of AP9 action is supported by data from β-arrestin-2-knockout animals. The absence of AP9 protective effect in mice lacking β-arrestin-2 indicates that AP9 can trigger β-arrestin-2-dependent pathways. β-Arrestin-2 recruits many effector proteins and provides G-protein-independent signaling via the mechanism of “biased agonism” (Figure 8) (Soh and Trejo, 2011; Griffin et al., 2015; Roy et al., 2016). It was previously shown that the localization of PAR1 in caveolae and its association with β-arrestins are necessary for activation of Rac1, which is a key participant in APC-mediated cytoprotective cascades (Soh and Trejo, 2011).

## Conclusion

In conclusion, the new PAR1 agonist peptide (NPNDKYEPF-NH_2_) demonstrates the APC-like protective effects in the mouse model of PT-induced brain ischemia. We suggest that AP9 action is mediated by its effects on the neurovascular unit and brain cells. The molecular mechanism of AP9 action is similar to that of APC. The activation of PAR1 by AP9, as well as APC-mediated PAR1 cleavage induce β-arrestin-2-dependent cytoprotective pathways. Our study shows the potential strategy to the treatment of ischemic brain injury based on a new class of peptide neuroprotectors.

## Data Availability Statement

The datasets generated for this study are available on request to the corresponding author.

## Ethics Statement

The animal study was reviewed and approved by the Bioethics Committee of Lomonosov Moscow State University.

## Author Contributions

MGa performed the research, analyzed the data, and wrote the manuscript. EK performed the histological research. MGu conducted the MRI studies. MS performed the peptide synthesis. LG designed the research and edited the manuscript. All authors contributed to the manuscript revision and read and approved the submitted version.

## Conflict of Interest

The authors declare that the research was conducted in the absence of any commercial or financial relationships that could be construed as a potential conflict of interest.

## Footnotes

1 http://www.randomization.com
