# Supplementary material for: New PAR1 Agonist Peptide Demonstrates Protective Action in a Mouse Model of Photothrombosis-Induced Brain Ischemia
Source: Front Neurosci. 2020 May 19;14:335. doi: 10.3389/fnins.2020.00335 (PMC7273131; doi:10.3389/fnins.2020.00335)
Supplement: Supplementary file 1 [file Data_Sheet_1.PDF]

Supplementary Figures

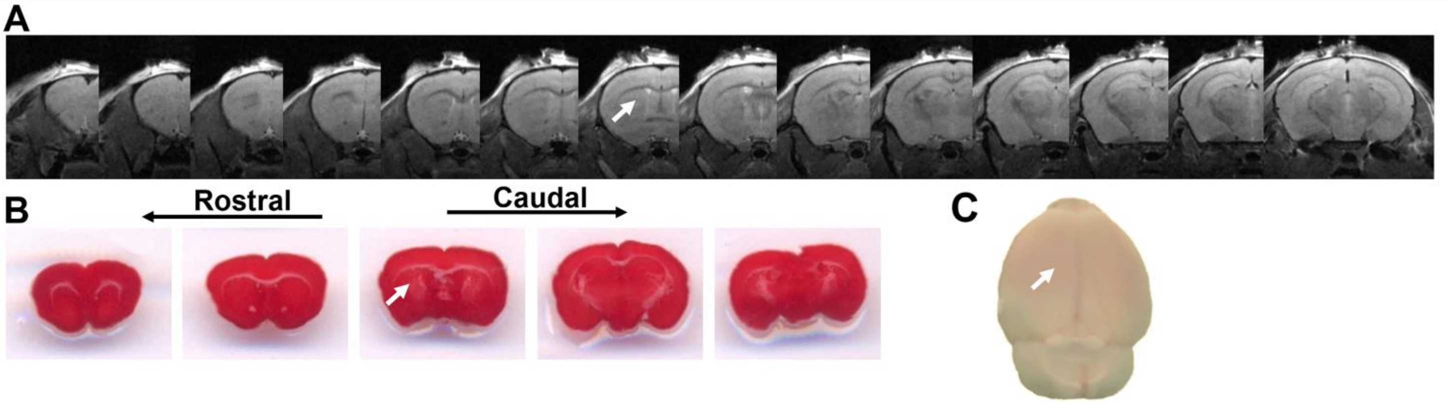

Supplementary Figure 1. (A) Representative MRI, **(B)** TTC staining and **(C)** Evans blue dye administration data obtained from sham-operated mice after the use of high-intensity laser irradiation (arrows indicate laser irradiated hemisphere).

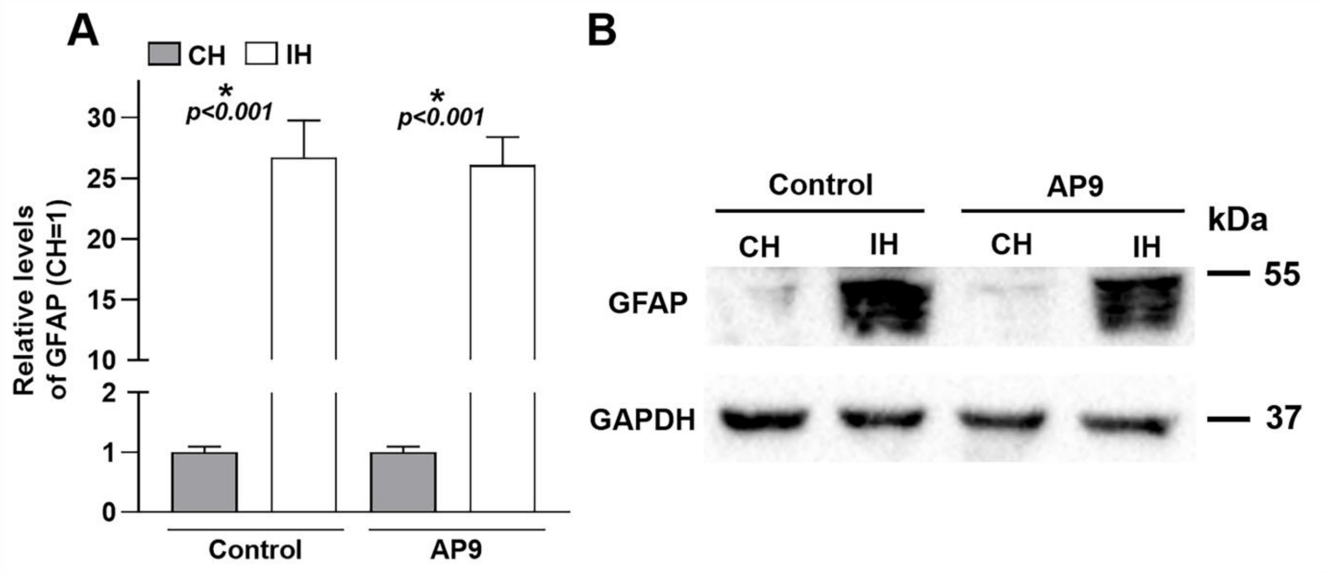

Supplementary Figure 2. GFAP expression 96 h post-thrombosis in control animals and AP9 treated mice: **(A)** data of Western blotting analysis and **(B)** representative blots of control and AP9 treated groups (double injection of AP9, 20 mg/kg; one-way ANOVA with Sidak correction for multiple comparisons, * – as compared to CH).
